# Environmental mutations in the Campo focus challenge elimination of sleeping sickness transmission in Cameroon

**DOI:** 10.1111/mve.12579

**Published:** 2022-05-20

**Authors:** Tito Tresor Melachio Tanekou, Calmes Ursain Bouaka Tsakeng, Inaki Tirados, Steve J. Torr, Flobert Njiokou, Alphonse Acho, Charles Sinclair Wondji

**Affiliations:** ^1^ Centre for Research in Infectious Diseases (CRID) Yaoundé Cameroon; ^2^ Department of Biological Sciences, Faculty of Science University of Bamenda Bamenda Cameroon; ^3^ Department of Biochemistry, Faculty of Science University of Yaoundé I Yaoundé Cameroon; ^4^ Department of Vector Biology Liverpool School of Tropical Medicine Liverpool UK; ^5^ Department of Animal Biology and Physiology, Faculty of Science University of Yaoundé I Yaoundé Cameroon; ^6^ Programme National de Lutte contre la Trypanosomose Humaine Africaine (PNLTHA) Ministry of Public Health Yaoundé Cameroon

**Keywords:** animal trypanosomiases, human sleeping sickness, South Cameroon, tsetse flies, vector control

## Abstract

Sleeping sickness is still prevalent in Campo, southern Cameroon, despite the efforts of World Health Organization and the National Control Programme in screening and treating cases. Reducing disease incidence still further may need the control of tsetse vectors. We update entomological and parasitological parameters necessary to guide tsetse control in Campo. Tsetse flies were trapped, their apparent densities were evaluated as the number of flies captured per trap per day and mapped using GIS tools. Polymerase chain reaction based methods were used to identify their trypanosome infection rates. *Glossina palpalis palpalis* was the dominant vector species representing 93.42% and 92.85% of flies captured respectively during the heavy and light dry seasons. This species presented high densities, that is, 3.87, 95% CI [3.84–3.91], and 2.51, 95% CI [2.49–2.53] flies/trap/day in the two seasons. Moreover, 16.79% (of 1054) and 20.23% (of 1132 flies) were found infected with at least 1 trypanosome species for the 2 seasons respectively, *Trypanosoma congolense* being the most prevalent species, and *Trypanosoma*. *brucei gambiense* identified in 4 samples. Tsetse flies are abundant in Campo and present high trypanosome infection rates. The detection of tsetse infected with human trypanosomes near the newly created palm grove show workers' exposition. Tsetse densities maps built will guide vector control with ‘Tiny Targets’.

## Graphical abstract

- High densities of tsetse fly vectors of Human sleeping sickness and animal trypanosomoses are recorded in the transborder focus of Campo, South Cameroon.
- Around 20% of these flies are infected with trypanosome parasites, among which *Trypanosoma brucei gambiense*, the human parasite.
- Population activities and new development projects implemented in the area (plantations creation, roads…) increase human exposure to tsetse bites and disease transmission risk, and therefore, vector control is needed.

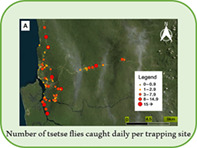

## INTRODUCTION

African trypanosomiases are vector‐borne tropical diseases caused by protozoan parasites of the genus *Trypanosoma* which are transmitted to susceptible hosts via the bite of blood‐sucking tsetse flies of the genus *Glossina* (Büscher et al., 2017). These diseases occur in 36 countries throughout sub‐Saharan Africa where about 60 million people, 55 million cattle and 70 million small ruminants are at risk (Cecchi & Mattioli, 2009; Kennedy, 2013). In humans, the disease is known as sleeping sickness or human African trypanosomiasis (HAT) while in livestock, it is called nagana or animal African trypanosomiasis (AAT). There are two forms of HAT. The more common form (98% of cases) occurs in West and Central Africa and is caused by *Trypanosoma brucei gambiense* (gHAT). The less common form (2% of cases) occurs in East and Southern Africa and is caused by *Trypanosoma brucei rhodesiense* (rHAT; Brun et al., 2010; Franco et al., 2020). Additionally, AAT is estimated to cost African agriculture USD 4.5 billion per year (Morrison et al., 2016) and therefore remains one of the main constraints for the development of livestock and agriculture in sub‐Saharan Africa (Diall et al., 2017).

Large outbreaks of HAT occurred in the first half of the 20th century and were largely brought under control in the 1960s, through large‐scale programs of active case detection and treatment of patients (World Health Organization [WHO], 1998). Following independence in many countries, the low incidence of HAT and political unrest led to reduced surveillance and neglect of the disease. As a consequence, a large‐scale resurgence occurred in the 1990s, with 45,000 new cases officially reported and around 500,000 estimated in large hard‐to‐reach remote areas (WHO, 1998). WHO's strategy for controlling gHAT has mainly relied on mass screening and treatment of cases implemented by National Control Programmes (NCPs) and has helped reducing by at least 90% the disease incidence over these three decades. However, this method often reaches less than 75% of the affected population (Tirados et al., 2015). In addition, potential animal reservoirs (Funk et al., 2013; Njiokou et al., 2006) may maintain the circulation of parasites even if all human cases are detected, which may make complete elimination of gHAT difficult (Funk et al., 2013; Njiokou et al., 2006; Simo et al., 2014). Thus, reducing vector populations and therefore human‐tsetse contact appear to be a complementary method to help stopping the transmission of the disease.

Recently, field experiments and mathematical models (Vale et al., 2015) have shown that the newly developed small screens of blue and black cloth netting impregnated with insecticide, known as ‘Tiny Targets’, can control tsetse vectors of gHAT effectively (Courtin et al., 2015; Tirados et al., 2020). These ‘Tiny Targets’ are easy to deploy, relatively cheap (Shaw et al., 2013), and have highly contributed to the recently reported decline of sleeping sickness (Mahamat et al., 2017; Ndung'u et al., 2020), with the lowest records of ~2164 new cases in 2016 (Kennedy & Rodgers, 2019), 977 in 2018 (Franco et al., 2020) and 565 in 2020 (WHO, 2021). With the vector control introduction, the WHO goal of eliminating gHAT as a public health problem was achieved in 2020 and the current goal is to achieve complete interruption of transmission by 2030.

Cameroon is among the countries that were targeted by the WHO for the elimination of transmission of sleeping sickness. Here, the most recent cases occurred in Campo, in the South forest Region, with around 7 cases/year between 2012 and 2018 (National Sleeping Sickness Control Programme records). In 2019, 20 new cases were detected, which implies, a prevalence of around 1%, that is, 100 times greater than the threshold for the elimination of the disease as a public health problem, as defined by WHO (i.e., 1 new case per 10,000 people). This increase may be related to the establishment of 70,000 hectares of the palm grove, and the arrival of 1000 employees working mainly in the forest biotope in close contact with tsetse flies. An increased risk of disease transmission was predicted by Simo et al. (2014), who listed the expected changes in habitat (deforestation and implantation of high structures, with prolonged presence of humans in at‐risk areas) as important risk factors for sleeping sickness elimination in Campo. This situation reinforced our will to show the importance of tsetse control in the area, and therefore prevent future increase of the incidence.

The present study is in line with one of the objectives of the PIIVeC Project (Partnership for Increasing the Impact of Vector Control—https://www.piivec.org/-), which aims to improve the policies of fighting vector‐borne diseases through the identification and implementation of appropriate actions to control their vectors. We report the current entomological situation in relation to Human and Animal trypanosomiases in Campo, by investigating tsetse fly vector distribution, the circulating trypanosome species and tsetse bloodmeal origins; particular attention is paid to ongoing environmental modifications occurring in the area. The results obtained will guide the tsetse control operation within the PIIVeC project.

## MATERIALS AND METHODS

### Study area

**FIGURE 1:**
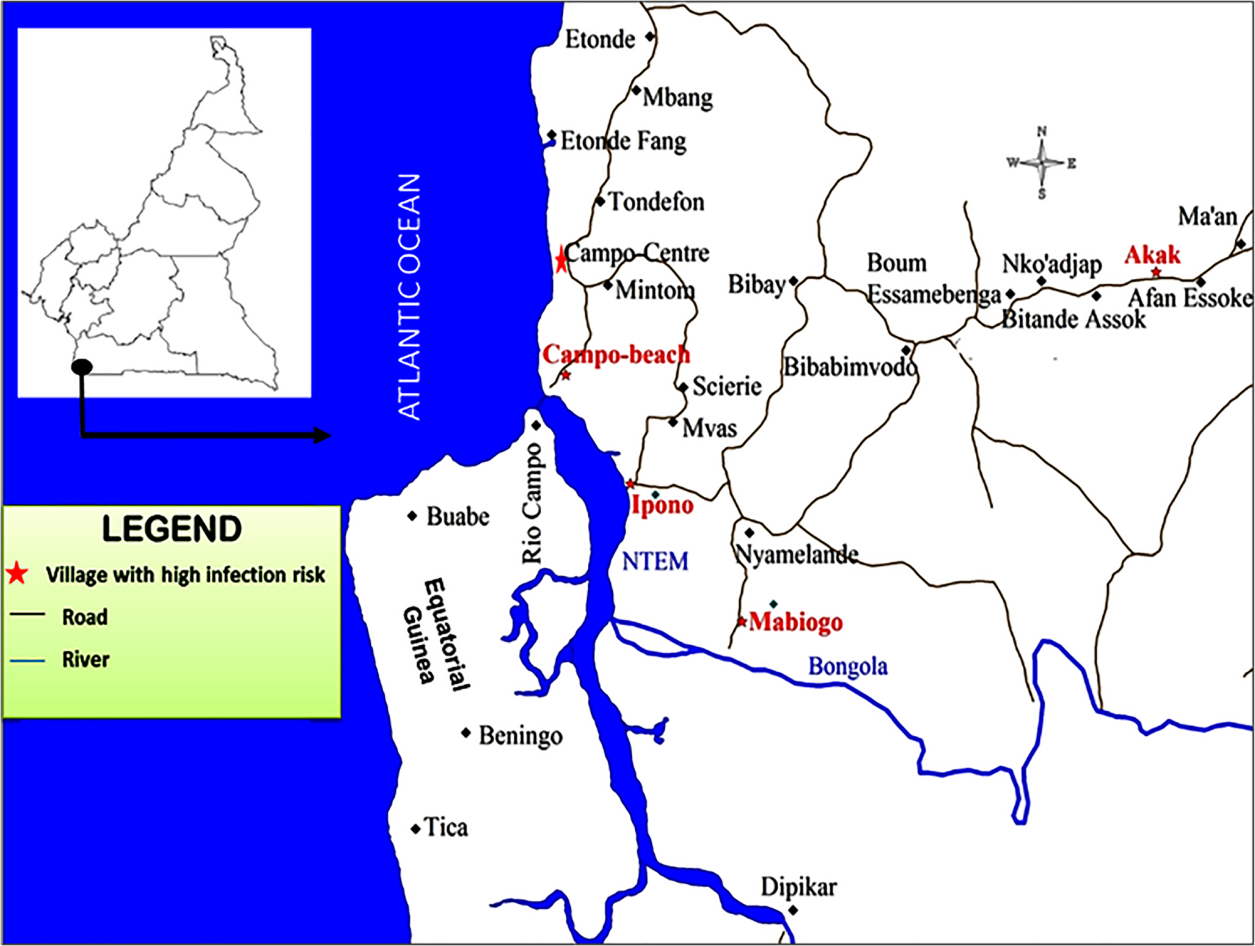
Campo, South Cameroon

Campo (2°22′N; 9°49′E) has been known as a sleeping sickness focus since 1902 (Penchenier et al., 1999). It is located on the Atlantic coast, near the border between Cameroon and Equatorial Guinea. It extends along the Ntem River (Figure 1) and is characterized by a climate of equatorial type, with four seasons which are a heavy and a light rainy seasons, and a heavy and a light dry seasons (the terms ‘heavy’ and ‘light’ are used to illustrate the intensity of rain with relatively low daily temperature for the rainy seasons, or almost absence of rain with relatively high temperature for the dry seasons). The main activities of Campo inhabitants are fishing, hunting and farming. The region has a dense hydrographic network with several rivers, swampy areas, marshes, and a large mangrove along the Ntem River. The gHAT focus in Campo is located near the Campo/Maan national park, where diverse wild fauna are reported (Njiokou et al., 2006) since its establishment in 1932. Across the River Ntem, important population movements occur between Campo Beach (Cameroon) and Rio Campo (Equatorial Guinea) for economic and familial purposes that may impact the epidemiology of HAT (Simo et al., 2014). Campo is a hypo‐endemic sleeping sickness focus where five tsetse species and sub‐species can be found, namely, *Glossina palpalis palpalis*, *G*. *pallicera*, *G*. *caliginea*, *G*. *nigrofusca* and *G*. *tabaniformis*; *G*. *palpalis palpalis* which is the HAT vector in the area is the most abundant (Grébaut et al., 2016; Simo et al., 2008).

### Entomological surveys

Two entomological surveys were conducted in heavy and light dry seasons (December 2018 and July 2019 respectively). Tsetse flies were collected using pyramidal traps (Gouteux & Lancien, 1986) placed in biotopes where tsetse are likely to concentrate: water points, rivers banks, behind dwellings, along the roads and farmlands. The trap positions were geo‐referenced with a global positioning system. The trapping points were selected according to the results obtained in a similar preliminary survey done in the same area in 2012 (Grébaut et al., 2016). Catch results in the selected sites were representative of the abundance and distribution in the entire surveyed area. Tsetse flies were collected once a day for two to four consecutive days, identified morphologically up to subspecies (Pollock, 1982), sex and teneral‐non teneral status (i.e., if they had taken their first bloodmeal or not), and preserved individually in Eppendorf tubes containing 95% ethanol. Once in the laboratory, these microtubes were stored at −20°C until DNA extraction.

### Determination of trypanosome infection rate in tsetse flies

#### DNA extraction

Prior to DNA extraction, fly heads were separated from the rest of the body, for further detection of mature *Trypanosoma congolense* or *Trypanosoma*. *vivax* infections. The DNA was extracted from tsetse fly heads and bodies using the LIVAK protocol (Livak, 1984). The tubes containing individual fly bodies or heads were left open at room temperature for evaporating the alcohol used for conservation. Five hundred microlitres of filtered and sterilized LIVAK buffer were introduced into each tube (LIVAK: 1.6 ml NaCl 5 M; 5.48 g Sucrose; 1.57 g Tris; 10.16 ml EDTA (Ethylenediaminetetraacetic acid) 0.5 M; 2.5 ml 20% sodium dodecyl sulfate; distilled water to 100 ml total volume). The contents of each tube were crushed and homogenized using a tube pestle and the tubes were incubated in a water bath at 65°C for 30 min. Then, 70 μl of potassium acetate was added, followed by incubation on ice for 30 min and centrifugation at 13,500 rpm for 20 min. The aqueous upper phase containing the nucleic acids was transferred into new Eppendorf tubes. One millilitre of absolute ethanol was then added for precipitation of the nucleic acids; the tubes were homogenized and centrifuged at 13,500 rpm for 15 min. The pellet obtained was washed twice with 200 μl of 70% ethanol. The alcohol was completely removed, and the tubes were air‐dried for about 1 h. The pellet was finally suspended in 30 and 100 μl of sterile water respectively for heads and bodies and stored at −20°C for subsequent molecular analyses.

#### Molecular identification of circulating trypanosomes

Trypanosomes were detected by a nested polymerase chain reaction (PCR) as described by Desquesnes et al. (2001), amplifying the Internal transcribed spacer 1 of the rDNA, using primers TRYP18.2C (5′‐GCAAATTGCCCAATGTCG‐3′) and TRYP4R (5′‐GCTGCGTTCTTCAACGAA‐3′) and IRFCC (5′‐CCTGCAGCTGGATCAT‐3′) and TRYP5RCG (5′‐ATCGCGACACCTTGTG‐3′) for the first and second reaction respectively. Reactions were performed in a final volume of 20 μl consisting of 2 μl of TBE buffer 10X (10 mM Tris–HCl; 1,5 mM MgCl_2_, 50 mM KCl, pH 8.3), 0.56 μl of each primer (10 μM), 0.4 μl of dNTPs (10 mM), 0.2 μl of Taq DNA polymerase (5 U/μl), 14.28 μl H_2_O and 2 μl of DNA extract. In the negative control tube, the DNA was replaced by 2 μl of sterile water. The amplification program consisted of an initial denaturation step at 94°C for 5 min, followed by 30 amplification cycles each consisting of denaturation at 94°C for 30 s, primer hybridization at 58°C for 1 min, elongation at 72°C for 1 min and a final elongation step at 72°C for 5 min. The composition of the reaction medium, as well as the amplification program, were the same for both reactions except that amplicon for the first PCR was diluted 1/10 before being used as a template for the second PCR.

The DNA extracts from *T*. *brucei* s.l. positive samples were subjected to a specific nested‐PCR for *T*. *b*. *gambiense* identification. The first reaction was done using primers TsGP1 (5′‐GCTGCTGTGTTCGGAGAGC‐3′) and TsGP2 (5′‐GCCATCGTGCTTGCCGCTC‐3) as described by Radwanska et al. (2002) and the amplification product was diluted 1/10 and used as a template for the second reaction with another pair of primers (TgsGPs: 5′‐TCAGACAGGGCTGTAATAGCAAGC‐3′ and TgsGPas: 5′‐GGGCTCCTGCCTCAATTGCTGCA‐3′) described by Morrison et al. (2008). All the amplification products were resolved in 2% agarose gels, stained with Midori Green.

### Blood meal origin determination

The origin of the remaining tsetse bloodmeals was determined by the amplification of a portion of the Cytochrome b gene from DNA extracts from fly bodies, using the universal vertebrate primers CYTBF (5′‐CCATCCAACATCTCAGCATGATGAAAA‐3′) and CYTBR (5′‐GCCTCAGAATGATTTGD:\keerthanaTCCTCA‐3′). Amplification was carried out in a final volume of 20 μl consisting of 2 μl TBE buffer (10X), 0.4 μl of dNTPs (10 mM), 0.8 μl of each primer (10 μM), 1 μl of MgCL_2_ (25 mM), 0.08 μl Taq DNA polymerase (5 U/μl), 12.92 μl H_2_O and 2 μl DNA extract. The amplification program consisted of an initial denaturation step at 94°C for 3 min and 30 s, followed by 40 cycles each containing a denaturation step at 94°C for the 30s, a primer hybridization step at 58°C for 30s and an extension step at 72°C for 1 min, plus a final extension step at 72°C for 5 min.

Cytochrome b PCR positive products were purified, sequenced, and species identification was performed using the BLASTn algorithm in GenBank DNA Sequence Database (http://www.ncbi.nlm.nih.gov/).

### Data analysis

The relative abundance of each tsetse fly species was estimated as the number of flies of that species over the total number of flies captured.

Catches per day and trap were normalized following a log transformation (e.g., $y_{i}=\log(x_{i}+1$)). The arithmetic mean of the transformed catches was obtained and back‐transformed to provide the apparent fly density per trap and per day (ADT), as in the following equation: $\mathrm{ADT}=10^{\bar{y}}-1$. These ADT data were mapped using the software QGIS 3.16 (QGIS Development Team, 2020).

Trypanosome infection rates in tsetse were computed as the percentage of flies found with trypanosome DNA over the total number of flies processed. A Chi‐square test was used to compare the infection rates of different trypanosome species between sampling periods, using the programme R with the significance threshold set at 0.05.

## RESULTS

### Relative abundance of tsetse species in Campo

**FIGURE 2:**
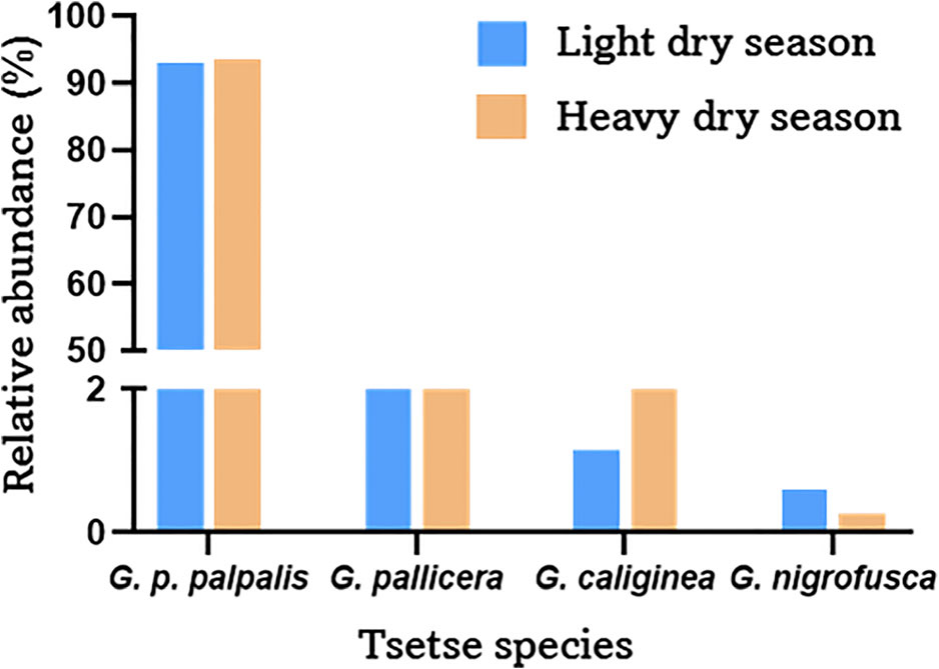
Relative abundance of tsetse fly species in Campo

A total of 1915 and 1300 tsetse flies were caught using 100 and 99 pyramidal traps, in heavy and light dry seasons respectively. In those captures, four tsetse fly species or sub‐species were clearly identified, namely *Glossina palpalis palpalis*, *G*. *pallicera*, *G*. *caliginea* and *G*. *nigrofusca*. *G*. *palpalis palpalis*, which is the sub‐species responsible for the transmission of the human sleeping sickness in Campo, was largely dominant, with a relative abundance of 93.42% and 92.85% for the two seasons (Figure 2). Regarding the other species present, *G*. *pallicera* accounted for 3.82% and 5.31%, *G*. *caliginea*, 2.45% and 1.15%, and *G*. *nigrofusca*, 0.26% and 0.62% for heavy and light dry seasons, respectively. For *G*. *palpalis palpalis*, although more females were caught in the light dry season (i.e., 61% of catches), the sex ratio was balanced in the heavy dry season; the teneral flies accounted for 5.07% and 5.85% respectively for the two seasons (Tables S1 and S2).

### *Apparent densities of* Glossina palpalis palpalis *in Campo*

**FIGURE 3:**
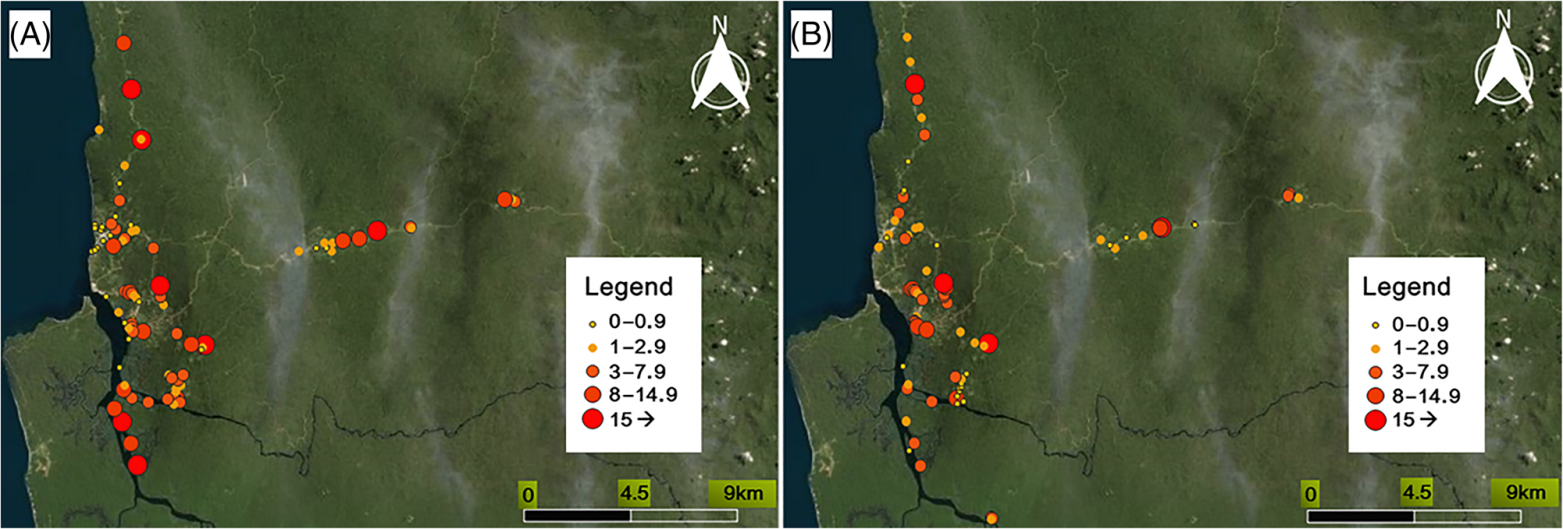
Map of the distribution of the apparent density per trap of *Glossina palpalis palpalis* (ADT) during December 2018 heavy dry season (a) and July 2019 light dry season (b) in Campo

The major vector of the Human sleeping sickness in Cameroon, *G*. *palpalis palpalis*, displayed high densities in the different sampling points in Campo (Figure 3), with mean ADT values of 3.87, 95% CI [3.84–3.91], and 2.51, 95% CI [2.49–2.53] flies/trap/day in heavy and light dry seasons respectively.

### Trypanosome infections in tsetse flies

#### Heavy dry season

After PCR‐based analyses of 1054 randomly selected flies of the sub‐species *G*. *palpalis palpalis* captured in December 2018, we identified 177 flies (16.79%) harbouring at least one trypanosome species in their midguts (Table 1). The most frequent species was *T*. *congolense* (15.37%), followed by *T*. *brucei* s.l. (1.52%), *T*. *vivax* (0.47%) and *T*. *simiae* (0.47%). Among the 177 tsetse flies with trypanosome infections, 11 (6.21%) were mixed infections, including 5 double infections with *T*. *congolense* and *T*. *brucei brucei*, 1 of *T*. *congolense* and *T*. *vivax*, 1 of *T*. *b*. *brucei* and *T*. *vivax* and 4 of *T*. *congolense* and *T*. *simiae*. Also, 40 (22.60%) of those infected flies contained mature infections, with trypanosome DNA detected in their mouthparts (heads), and all these were of the species *T*. *congolense*.

**TABLE 1:** Trypanosome infection rates in *Glossina palpalis palpalis* samples

	Sample size	TC (%)	95% CI	TB (%)	95% CI	TV (%)	95% CI	TS (%)	95% CI	Tbg (%)	Total (%)	95% CI	
December 2018	1054	162 (15.37)	[13.2–17.6]	16 (1.52)	[0.8–2.4]	5 (0.47)	[0.1–1.1]	5 (0.47)	[0.1–1.1]	1 (0.09)	177 (16.79)	[15.5–20.2]	
July 2019	1132	137 (12.10)	[10.2–14.1]	85 (7.51)	[6–9.2]	29 (2.56)	[1.7–3.6]	2 (0.18)	[0–0.6]	3 (0.26)	229 (20.23)	[19.9–24.8]	
*p*‐value		0.03		<0.001		<0.001		0.39			0.01		

Abbreviations: TB, *Trypanosoma brucei* s. l.; Tbg, *Trypanosoma brucei gambiense*; TC, *Trypanosoma congolense*; TS, *Trypanosoma simiae*; TV, *Trypanosoma vivax*.

*T*. *brucei gambiense*, the human parasite responsible for the sleeping sickness, was identified in one sample among the *T*. *brucei* s.l. infections.

#### Light dry season

A total of 1132 flies captured during the light dry season (July 2019) were tested and 229 (20.23%) midguts were found infected by at least one *Trypanosoma* species. As of December 2018, *T*. *congolense* (12.10%) was the most common, followed by *T*. *brucei* s.l. (7.51%), *T*. *vivax* (2.56%) and *T*. *simiae* (0.18%).

Among the 229 tsetse flies with trypanosome infections, 23 (10.04%) were mixed infections, including 18 double infections of *T*. *congolense* and *T*. *brucei*, 4 of *T*. *congolense* and *T*. *vivax* and 1 triple infection of *T*. *congolense*, *T*. *brucei* and *T*. *vivax*. No matured infection was identified in these infected flies.

Out of the 85 samples infected with *T*. *brucei* s.l., 3 (1.31% of the total *Trypanosoma* infections, and therefore, 3.53% of *T*. *brucei* s.l.) were identified as *T*. *brucei gambiense*.

Overall, trypanosome infection rates were significantly different between trypanosome species and between the two sampling periods (Table 1). Of the 100 and 99 traps deployed in December 2018 and July 2019, 58 (58%) and 80 (80.8%) caught at least one tsetse fly infected by trypanosomes respectively. Although the traps that caught at least one infected fly were widely distributed in different areas, infection rates were significantly different between villages (*p* < 0.001, data not shown) suggesting that the transmission patterns may vary according to their locations.

### Determination of tsetse blood meal sources

Vertebrates' cytochrome B DNA was successfully amplified in 40 and 45 *G*. *palpalis palpalis* samples from December 2018 and July 2019 trips respectively. After sequencing and aligning to reference databases (in Genbank), 2 out of 40 (from December) aligned perfectly with Human DNA, showing that the tsetse concerned fed on humans. The 38 remaining from that trip, plus the 45 of July corresponded to four different profiles but did not align with any known vertebrate.

## DISCUSSION

Tsetse control can contribute towards WHO's goal of elimination of transmission of the sleeping sickness. In the present study, we give an overview of the entomological situation of gHAT prior to a vector control intervention planned in Campo HAT focus.

### The high density of tsetse flies highlights the significant entomological risk in Campo

During two entomological surveys, 3215 tsetse flies were collected, and four tsetse species were identified, namely *G*. *palpalis*, *G*. *pallicera*, *G*. *nigrofusca* and *G*. *caliginea*, plus two individuals belonging to a fifth species that could not be identified morphologically using the key we had. These results are in agreement with those reported in previous studies, indicating that ecological conditions of these biotopes remain favourable to the development of these taxa (Farikou et al., 2010a; Grébaut et al., 2016; Simo et al., 2008). *G*. *palpalis*, which is the vector of sleeping sickness in Campo, was the predominant species captured, whatever the sampling period or the sampling point (around 93%). This result is consistent with previous studies in the same area (Grébaut et al., 2016; Simo et al., 2008). The predominance of this species may be due to the important modifications of the biotopes with demographic changes during the development (Cecchi et al., 2009). In fact, *G*. *palpalis palpalis* which is more anthropophilic persists in human‐degraded areas compared to other species that are more zoophilic (Simo et al., 2008). The deforestation and the creation of farmlands like the ongoing 70,000 hectares palm grove near the Campo natural game reserve or wood exploitations in Campo, and other projects like the autonomous port at Kribi (around 35 km from Campo) or the hydroelectric dam at Memvele attracted people and increased the local population by 20% in the three past years; the settlement of these populations and creation of farmlands made the Campo environment more anthropized and thus, suitable for colonization by *G*. *palpalis palpalis* (Simo et al., 2014); it is important to note that records of the years 1974 (Eouzan et al., 1974) or 2001 (Mbida Mbida, Unpublished results) show *G*. *palpalis* relative abundance not exceeding 65%. Moreover, this study has shown high apparent densities for this species in the two seasons, that is, 3.87 and 2.59 flies/trap/day. These densities are 2–3 times greater than the ones observed in 2012 in the same area (Grébaut et al., 2016). This observation indicates the high potential risk of contact between human and tsetse flies, and thus an increased disease transmission risk. Also, this result confirms that the deep ongoing forest degradation and implantation of agricultural‐related activities are favourable to the development of the anthropophilic *G*. *palpalis palpalis*, which is, unfortunately, responsible for sleeping sickness transmission in the area.

### The detection of trypanosome infected tsetse flies supports the circulation of these parasites in Campo

Molecular identification of trypanosomes revealed a high prevalence of the animal trypanosome species *T*. *congolense*, *T*. *brucei brucei*, *T*. *vivax* and *T*. *simiae*, and the presence of the human parasite, *T*. *brucei gambiense* in *G*. *palpalis palpalis* captured in Campo. These trypanosome species were already reported in tsetse captured in Campo by several authors (Grébaut et al., 2016; Ngambia Freitas et al., 2021; Simo et al., 2015; Tsagmo Ngoune et al., 2017), or in animals of the same area or from other trypanosomiases foci in Cameroon (Mamoudou et al., 2006; Nimpaye et al., 2011; Njiokou et al., 2004; Simo et al., 2006; Tanenbe et al., 2010). These results confirm the current circulation of trypanosomes in the Campo forest area of Cameroon. However, a significant difference was observed in trypanosome infection rates in flies between December 2018 and July 2019 (*p*‐value of 0.01). Indeed, *T*. *congolense* infection rate was smaller in July compared to December, whereas *T*. *brucei* and *T*. *vivax* rates were ~5 times higher in July. These results suggest that trypanosome circulation is dynamic over time and may be linked to vertebrate hosts present. In fact, a study conducted in Campo on the dynamics of potential wild tsetse hosts showed that although some wild animal species (*Cephalophus monticola*, *Cephalophus dorsalis*, *Atherurus africanus*, *Cricetomys emini*, *Nandinia binotata* and *Cercopithecus nictitans*) occur in all biotopes in all seasons, other (*C*. *monticola*, *Tragelaphus spekei* and *C*. *emini* for example) move across biotopes (from farmlands to forest near villages) according to the season (Massussi et al., 2009). Also, 22.6% of infected flies in December 2018 were carrying mature infections with *T*. *congolense* and none from July 2019 were found with mature infection; this result suggests that the latter were recently infected and did not have enough time to develop their parasites before being captured, meaning that flies' infection in the nature may follow cycles of hosts availability in their biotopes. In our study, the occurrence of mature trypanosome infections was certainly underestimated, since we did not dissect the fly salivary glands that are the localization of mature *T*. *brucei* infections. Among the four trypanosome species identified, *T*. *congolense* predominated. *T*. *congolense* is one of the trypanosome species widely distributed in forest areas like Campo, where it presents high infectivity to domestic animals (Bengaly et al., 2002; Nimpaye et al., 2011; Simo et al., 2013), but also, it has been reported in wild animals species of the area (Herder et al., 2002; Njiokou et al., 2004). Concerning *T*. *simiae*, they were detected with low infection rates in *G*. *palpalis palpalis*. This result corroborates the low prevalence of this parasite, previously reported in domestic and wild animals in Campo (Nimpaye et al., 2011; Njiokou et al., 2004); this parasite is a potential constraint for the implementation of pig rearing in the area due to its high virulence for pigs (Moloo et al., 1992); the low observed infection rate in tsetse may be indicative of the fact that infected animals die before having enough time for infecting the neighbouring tsetse flies, or other animals clearing the infection with their immune system (Bruce et al., 1913). The mixed trypanosome infections identified in tsetse flies reflect the presence of such infections in mammalian hosts living in the area. Thus, during the intake of a bloodmeal, tsetse flies can ingest one or more trypanosome species from an animal. These results are already reported in previous studies (Grébaut et al., 2016; Simo et al., 2015; Tchouomene‐Labou et al., 2013).

### The presence in tsetse flies of human bloodmeal and human trypanosome highlight the persistent risk of sleeping sickness transmission in Campo

Two human bloodmeals out of 85 were identified in *G*. *palpalis palpalis*; the others (belonging to 4 unique sequences, thus 4 different animals) could not be identified. This result suggests that on top of humans, tsetse flies feed on other vertebrates. In this study, unknown bloodmeal sources are likely to belong to wild animals, for which cytochrome b sequences are absent from the Genbank database used for their identification as already shown by Farikou et al. (2010b). No blood meal was identified from domestic animals in this study; this may be due to the fact that the tsetse flies used here mostly originate from forest biotopes, while domestic animal rearing is done in town where the tsetse are now scarce because of poor habitat suitability.

*T*. *brucei gambiense*, responsible for human sleeping sickness, was found in the villages of Mabiogo, Nazareth, Mvass and military surveillance posts on the border between Cameroon and Equatorial Guinea. These villages are those where sleeping sickness cases were detected these later years. In Mvass, the implementation of new farmland near a river with high tsetse density, and where young palm trees are produced for the palm grove, with more than 1000 employees in contact with the river daily, represents a great risk of disease transmission. Moreover, these villages bordered by mangroves with high tsetse densities are mainly located at the border between Cameroon and Equatorial Guinea, where human activities increase the risk of transborder transmission of sleeping sickness. Also, many soldiers guarding the border have been found infected and treated for sleeping sickness; their regular movement from Campo to other posts in northern Cameroon infested by tsetse vectors like *G*. *palpalis*, *G*. *fuscipes* and *G*. *tachinoides*, or to other countries like the Central African Republic for security issues clearly extend the risk of expanding the disease at a larger scale.

## CONCLUSION

Although development projects substantially improve the economy in developing countries, some may increase the risk of transmission of certain diseases like dams increasing the risk of schistosomiases, some crops like rice, the risk of malaria, and others the risk of sleeping sickness, and thus threaten their sustainability. Therefore, environmental risk assessments before implementation can help mitigating potential risks by taking necessary action. The results of the present study have provided an updated spatial distribution of the tsetse fly species *G*. *palpalis palpalis*, the vector of Human and animal trypanosomiases in the Campo focus of southern Cameroon. We showed a high level of trypanosome circulation in Campo, including the presence of the human parasite, *T*. *brucei gambiense* despite the efforts made by WHO and the National Sleeping Sickness Control Programme in cleaning the human reservoir of the parasite. The high densities of the tsetse vector revealed here threaten all those efforts of disease elimination. Our results also highlight many transmission risk factors, like the new plantation in which tsetse are found infected with human trypanosomes. The results generated and mapped in the current article are of high relevance for the design of an effective tsetse control operation with ‘Tiny Targets’.

## AUTHOR CONTRIBUTIONS

*Study design*: Tito Tresor Melachio Tanekou, Charles Sinclair Wondji and Steve J. Torr. *Field data collection*: Tito Tresor Melachio Tanekou, Calmes Ursain Bouaka Tsakeng, Inaki Tirados, Alphonse Acho. *Sample analyses*: Calmes Ursain Bouaka Tsakeng, Tito Tresor Melachio Tanekou. *Data analyses*: Calmes Ursain Bouaka Tsakeng, Inaki Tirados, Steve J. Torr, Tito Tresor Melachio Tanekou. *Manuscript preparation*: all authors. *General Supervision*: Charles Sinclair Wondji, Steve J. Torr and Flobert Njiokou.

## CONFLICT OF INTEREST

The authors declare that they have no conflict of interest.

## Supporting information


**Table S1**. Composition of tsetse fly species in Campo during the heavy dry season (December 2018).

**Table S2**. Composition of tsetse fly species in Campo during the light dry season (July 2019).

## Data Availability

Most of the data that support the findings of this study are presented in this paper and in supplementary files provided; other details are available from the corresponding author upon reasonable request.
