# Supplementary material for: Environmental mutations in the Campo focus challenge elimination of sleeping sickness transmission in Cameroon
Source: Med Vet Entomol. 2022 May 20;36(3):260–8. doi: 10.1111/mve.12579 (PMC10138755; doi:10.1111/mve.12579)
Supplement: Supplementary file 1 — Table S1. Composition of tsetse fly species in Campo during the heavy dry season (December 2018). [file MVE-36-260-s001.docx]

**Supplementary Table 1**: Composition of tsetse fly species in Campo during the heavy dry season (December 2018)

| Villages | Number captured | Num. *G. caliginea* | Num. *G. nigrofusca* | Num. *G. pallicera* | Num. *G. palpalis palpalis* | NI | Num. Teneral | Num. Traps | ADT (Flies/ Trap/Day) |
| --- | --- | --- | --- | --- | --- | --- | --- | --- | --- |
| Afan Essokie | 419 | 8 | 3 | 17 | 391 | 0 | 12 | 2 | 69.83 |
| Akak | 62 | 0 | 0 | 0 | 62 | 0 | 5 | 3 | 6.89 |
| Assok | 5 | 0 | 0 | 0 | 5 | 0 | 0 | 1 | 1.67 |
| Bokome-Centre | 7 | 3 | 0 | 0 | 4 | 0 | 0 | 4 | 0.58 |
| Campo-Beach | 120 | 8 | 0 | 5 | 107 | 0 | 5 | 6 | 6.67 |
| Campo-Ville | 17 | 0 | 0 | 0 | 17 | 0 | 1 | 3 | 1.89 |
| Paris soir | 1 | 0 | 0 | 0 | 1 | 0 | 0 | 1 | 0.33 |
| Snec | 14 | 0 | 0 | 0 | 14 | 0 | 0 | 1 | 4.67 |
| Doumassi | 38 | 9 | 0 | 1 | 28 | 0 | 4 | 1 | 12.67 |
| Enyengenamanga | 2 | 0 | 0 | 1 | 1 | 0 | 0 | 2 | 0.33 |
| Ipono | 82 | 4 | 0 | 4 | 74 | 0 | 0 | 6 | 4.56 |
| Ipono-Sierie | 28 | 0 | 0 | 0 | 28 | 0 | 3 | 2 | 4.67 |
| Itonde-Mer | 8 | 0 | 0 | 0 | 8 | 0 | 1 | 2 | 1.33 |
| Itonde-Afan Essokie | 75 | 2 | 0 | 3 | 70 | 0 | 6 | 1 | 25 |
| Itonde-Ecole | 77 | 2 | 0 | 1 | 74 | 0 | 3 | 3 | 8.56 |
| Itonde-Mbala Mbala) | 46 | 1 | 0 | 7 | 38 | 0 | 4 | 1 | 15.33 |
| Itonde-Mbanga | 3 | 0 | 0 | 0 | 3 | 0 | 0 | 1 | 1 |
| Itonde-Mehibao | 22 | 0 | 0 | 2 | 20 | 0 | 0 | 1 | 7.33 |
| Itonde-Washinton | 2 | 0 | 0 | 0 | 2 | 0 | 0 | 1 | 0.67 |
| Maan | 7 | 0 | 0 | 0 | 7 | 0 | 0 | 1 | 2.33 |
| Mabiogo | 183 | 3 | 1 | 18 | 160 | 1 | 9 | 13 | 4.69 |
| River Ntem banks | 261 | 4 | 0 | 0 | 257 | 0 | 27 | 15 | 5.76 |
| Mintomb | 20 | 0 | 0 | 1 | 19 | 0 | 1 | 2 | 3.33 |
| Monakak | 2 | 0 | 0 | 0 | 2 | 0 | 0 | 1 | 0.67 |
| Mvass | 139 | 2 | 0 | 2 | 135 | 0 | 5 | 10 | 4.63 |
| Nazareth | 160 | 0 | 1 | 8 | 151 | 0 | 10 | 6 | 8.89 |
| Nkoelong | 76 | 0 | 0 | 3 | 73 | 0 | 0 | 4 | 6.33 |
| Nkouandjap | 34 | 1 | 0 | 0 | 33 | 0 | 1 | 5 | 2.27 |
| Okanbiloun | 5 | 0 | 0 | 0 | 5 | 0 | 0 | 1 | 1.67 |
| Total | **1915** | **47**  **(2.45%)** | **5**  **(0.26%)** | **73**  **(3.82%)** | **1789**  **(93.42%)** | **1**  **(0.05%)** | **97 (5.07%)** | **100** |  |

Num.: Number; *G.*: *Glossina*; NI: non identified; ADT: Apparent density per trap
