# Supplementary material for: Environmental mutations in the Campo focus challenge elimination of sleeping sickness transmission in Cameroon
Source: Med Vet Entomol. 2022 May 20;36(3):260–8. doi: 10.1111/mve.12579 (PMC10138755; doi:10.1111/mve.12579)
Supplement: Supplementary file 2 — Table S2. Composition of tsetse fly species in Campo during the light dry season (July 2019). [file MVE-36-260-s002.docx]

**Supplementary Table S2**: Composition of tsetse fly species in Campo during the light dry season (July 2019)

| Villages | Number captured | Num. *G. caliginea* | Num. *G. nigrofusca* | Num. *G. pallicera* | Num. *G. palpalis palpalis* | NI | Num. Teneral | Num. Traps | ADT (Flies/ Trap/Day) |
| --- | --- | --- | --- | --- | --- | --- | --- | --- | --- |
| Afan Essokie | 270 | 3 | 2 | 21 | 244 | 0 | 8 | 2 | 45 |
| Akak | 7 | 0 | 0 | 1 | 6 | 0 | 0 | 3 | 0,77 |
| Assok | 3 | 0 | 0 | 0 | 3 | 0 | 0 | 1 | 1 |
| Bokome-Centre | 6 | 0 | 0 | 0 | 6 | 0 | 0 | 2 | 1 |
| Campo-Beach | 86 | 2 | 0 | 1 | 83 | 0 | 3 | 6 | 4,77 |
| Campo-Ville | 1 | 0 | 0 | 0 | 1 | 0 | 0 | 4 | 0,08 |
| Snec | 4 | 0 | 0 | 0 | 4 | 0 | 0 | 2 | 0,66 |
| Ipono | 128 | 4 | 0 | 0 | 124 | 0 | 16 | 8 | 5,33 |
| Itonde | 29 | 0 | 0 | 0 | 29 | 0 | 1 | 7 | 1,38 |
| Itonde-Afan Essokie | 57 | 1 | 0 | 1 | 55 | 0 | 8 | 2 | 9,5 |
| Itonde-Ecole | 22 | 0 | 0 | 0 | 22 | 0 | 0 | 2 | 3,66 |
| Itonde-Mbala Mbala | 6 | 0 | 0 | 0 | 6 | 0 | 0 | 1 | 2 |
| Itonde-Mbanga | 1 | 0 | 0 | 0 | 1 | 0 | 0 | 1 | 0,33 |
| Itonde-Mehibao | 12 | 0 | 0 | 0 | 12 | 0 | 1 | 2 | 2 |
| Maan | 4 | 0 | 0 | 0 | 4 | 0 | 0 | 2 | 0,66 |
| Mabiogo | 85 | 1 | 3 | 16 | 65 | 0 | 1 | 11 | 2,575 |
| River Ntem banks | 136 | 1 | 0 | 9 | 126 | 0 | 16 | 16 | 2.83 |
| Mintomb | 21 | 1 | 0 | 0 | 20 | 0 | 0 | 3 | 2,33 |
| Mvass | 166 | 0 | 0 | 13 | 153 | 0 | 11 | 8 | 6,91 |
| Nazareth | 200 | 2 | 3 | 7 | 187 | 1 | 9 | 7 | 9,52 |
| Nkoelong | 35 | 0 | 0 | 0 | 35 | 0 | 2 | 4 | 2,91 |
| Nkouandjap | 17 | 0 | 0 | 0 | 17 | 0 | 0 | 3 | 1,88 |
| Okanbiloun | 4 | 0 | 0 | 0 | 4 | 0 | 0 | 2 | 0,66 |
| Total | **1300** | **15 (1.15%)** | **8**  **(0.62%)** | **69 (5.31%)** | **1207 (92.85%)** | **1 (0.07%)** | **76 (5.85%)** | **99** |  |

Num.: Number; *G.*: *Glossina*; NI: non identified; ADT: Apparent density per trap
